# Vascular Involvement in Thymic Epithelial Tumors: Surgical and Oncological Outcomes

**DOI:** 10.3390/cancers13133355

**Published:** 2021-07-04

**Authors:** Giovanni M. Comacchio, Andrea Dell’Amore, Maria Carlotta Marino, Michele Dario Russo, Marco Schiavon, Marco Mammana, Eleonora Faccioli, Giulia Lorenzoni, Dario Gregori, Giulia Pasello, Giuseppe Marulli, Federico Rea

**Affiliations:** 1 Thoracic Surgery and Lung Transplant Unit, University Hospital of Padua, 35128 Padua, Italy; giovannimaria.comacchio@aopd.veneto.it (G.M.C.); andrea.dellamore@unipd.it (A.D.); mariacarlotta.marino@aopd.veneto.it (M.C.M.); micheledario.russo@aopd.veneto.it (M.D.R.); marco.schiavon@unipd.it (M.S.); marco.mammana@aopd.veneto.it (M.M.); faccioli.eleonora@gmail.com (E.F.); 2 Unit of Biostatistics, Epidemiology and Public Health, University of Padua, 35131 Padua, Italy; giulia.lorenzoni@unipd.it (G.L.); dario.gregori@unipd.it (D.G.); 3 Oncology 2, Istituto Oncologico Veneto IOV—IRCCS, 35128 Padua, Italy; giulia.pasello@iov.veneto.it; 4 Thoracic Surgery Unit, University Hospital of Bari, 70124 Bari, Italy; giuseppe.marulli@uniba.it

**Keywords:** thymic tumor, vascular involvement, surgery, tumor recurrence

## Abstract

**Simple Summary:**

The involvement of mediastinal great vessels is common in advanced stage thymic tumors, which makes their surgical resection challenging. Moreover, the impact of vascular involvement on the oncological prognosis is still unclear. The aim of our retrospective, single-center study is to investigate surgical and oncological in a population of patients operated for advanced stage thymic tumors, dividing them in two groups according to the presence or absence of vascular involvement. We demonstrated that resection of thymic tumors with vascular involvement can be performed with optimal surgical results in a high-volume center and that the involvement of the great vessels seems to be associated with a higher recurrence rate, without affecting long-termsurvival.

**Abstract:**

Background: The involvement of mediastinal great vessels is common in advanced stage thymic tumors, which makes their surgical resection challenging. Moreover, the impact of vascular involvement on the oncological prognosis is still unclear. The aim of this study is to investigate the surgical and oncological outcomes and the impact of vascular involvement in a population of patients operated for advanced stage thymic tumors. Methods: A retrospective analysis on four hundred and sixty-five patients undergoing resection for advanced stage (Masaoka III–IV) thymic tumors in a single high-volume center was performed. One hundred forty-four patients met the inclusion criteria and were eligible for the study. Patients were divided in two groups according to the presence or absence of vascular involvement. Results: the two groups did not differ for the baseline characteristics and showed comparable surgical outcomes. Vascular involvement was not associated with worse overall survival but with an increased recurrence rate (*p* = 0.03). Multivariable analysis demonstrated a higher risk of recurrence in patients without R0 resection (HR 0.11, 0.02–0.54, *p* = 0.006) and with thymic carcinoma (HR 2.27, 1.22–4.24, *p* = 0.01). Conclusions: resection of thymic tumors with vascular involvement can be performed with optimal surgical results in a high volume center. From the oncological point of view, the involvement of the great vessels seems to be associated with a higher recurrence rate without affecting long-term survival.

## 1. Introduction

Thymic epithelial tumors (TET), although rare, are the most common neoplasms arising in the anterior mediastinum. The involvement of the surrounding structures is a common feature in advanced stages of the disease, while pleural implants are the most frequent site of metastasis, which can already be present at the time of diagnosis.

Surgery is still considered the “gold standard” in the treatment of TET when a radical resection is deemed feasible. In advanced stages however, a complete macroscopical resection is not always achievable. Therefore, no common agreement yet exists on which is the best therapeutic strategy to adopt for Masaoka stage III–IV tumors, but multimodality strategies with various combinations of chemotherapy, surgery, and radiotherapy seem to improve outcomes [1].

Different reports showed satisfying results in the case of the surgical resection of TET associated with the resection and reconstruction of the mediastinal great vessels, mainly the superior vena cava and innominate veins, involved by thymic tumors. However, the experiences reported in the literature show a wide range of techniques and reconstructive materials [2].

From an oncological point of view, the impact of the vascular involvement on overall and relapse-free survival is still unclear. Different studies reported a negative impact of vascular invasion in terms of survival or recurrence risk, while other large series found that there were no prognostic differences when analyzing patients according to the involved organ [3,4,5,6,7].

The aim of this study is to investigate the surgical outcomes and the oncological impact of vascular involvement in a population of patients operated for advanced stage thymic tumors.

## 2. Materials and Methods

A retrospective analysis of a prospectively maintained database on patients undergoing surgical resection of thymic epithelial tumors at the Thoracic Surgery Division of Padua, between 1990 and December 2020 was performed.

Inclusion criteria for this study were as follows: pathological confirmation of thymic epithelial tumor (thymoma or thymic carcinoma); pathological Masaoka stage III or IV; complete resection of the tumor (R0 or R1 resection); complete basic and follow-up information available.

Eligible patients for the study were divided into two groups according to the presence of vascular involvement. Patients who underwent vascular resection but with absence of pathologically confirmed involvement of the vessel were considered in the group without vascular involvement.

Preoperative work-up included medical history and physical examination, complete biochemical profile, bronchoscopy, total body computed tomography (CT) scan in all patients, chest magnetic resonance imaging (MRI) in selected patients with suspicion of vascular invasion and, since 1998, positron emission tomography (PET) scan.

Histological diagnosis of thymic tumor was reviewed and confirmed by pathologists according to the World Health Organization (WHO) classification [8], while pathological staging was based on the WHO TNM and Masaoka-Koga classification [9,10].

All cases were evaluated in a multidisciplinary setting and patients for whom an upfront surgical resection was not deemed feasible underwent a platinum-based induction chemotherapy. After chemotherapy, the treatment’s response was routinely evaluated by CT- or PET-CT scan. Patients without disease progression underwent surgery within 8 weeks from the end of the induction treatment.

The surgical approach consisted in the complete excision of the thymoma, the entire thymic gland and the surrounding mediastinal fat. An extended resection of surrounding infiltrated organs (e.g., lung, pericardium, pleura, vessels) was performed to obtain a complete macroscopic resection.

The type of vascular resection and reconstruction was based on the site and the depth of tumor invasion evaluated during the surgical procedure.

In case of limited partial infiltration, after the dissection of the vessel, a tangential clamp of the vessel can be sufficient to remove the involved portion of the wall. If the remaining lumen is more than 50%, a direct continuous suture with 4–5/0 polypropylene can be performed. To avoid stenosis or kinking of the vessel, the continuous suture is performed perpendicularly to the long axis of the vessel.

In case of a larger defect, the vessel repair is performed using a patch technique. For reconstruction, an autologous or heterologous pericardial patch is used accordingly to the surgeon’s preferences.

In case of circumferential resection, a reconstruction with a vascular conduit is necessary. We generally use a ringed polytetrafluoroethylene (PTFE) conduit. We always perform firstly the proximal anastomoses in a continuous fashion with 4–5/0 polypropylene and then, with the same technique, the distal one. All but one of the patients underwent vascular conduit reconstruction by the cross-clamping technique or using a veno-venous shunt between the internal jugular vein and femoral vein, with continuous monitoring of the internal jugular vein pressure. In one case, we used a cardiopulmonary bypass because of a neoplastic thrombus protruding in the right atrium.

**Figure 1:**
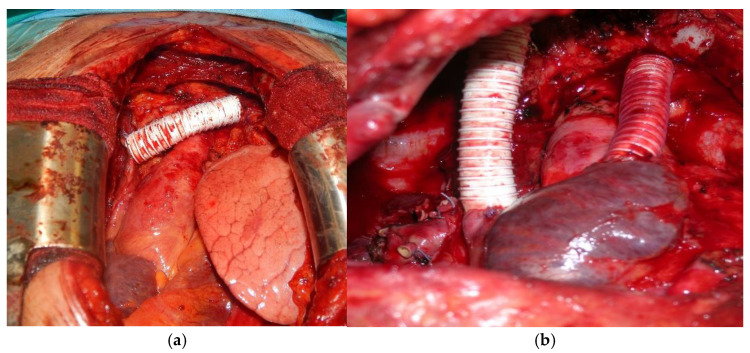
(**a**) Prosthetic replacement of the LIV; (**b**) Bilateral replacement of the innominate veins and of the SVC: prosthetic conduit between the RIV and the origin of the SVC and between the LIV and the right auricle.

According to the location and the extent of vessel involvement, different approaches were applied for reconstruction, such as isolated left innominate vein (LIV) replacement, LIV—superior vena cava (SCV), right innominate vein (RIV)—SCV, innominate veins—right atrium double bypass (Figure 1a,b). In case of involvement of one or both innominate veins, restoring the patency of both vessels was not always deemed necessary, especially in case of chronic obstruction and where collateral circles were already present.

The patients were followed up with a chest CT scan four months after operation, then every six months for the first two years and finally every year for at least 10 years. Follow-up information was obtained from clinical visits and phone interviews.

Recurrence after resection was defined as local recurrence, regional recurrence (i.e., pleural, or pericardial) or distant recurrence (extrathoracic relapse or intraparenchymal pulmonary nodules) according to ITMIG proposal [11].

Overall survival (OS) was defined as the time interval from surgery to the date of the last follow-up or death for all causes. Disease free interval (DFI) was defined as the time interval from surgery to the date of first recurrence.

### Statistical Analysis

Descriptive statistics were reported as I quartile/median/III quartile for continuous variables and percentages (absolute numbers) for categorical variables. Wilcoxon and chi-squared tests were performed to compare the distribution of continuous and categorical variables, respectively, in the descriptive analysis.

Survival distribution was evaluated using the Kaplan–Meier approach, while disease recurrence was evaluated using cumulative incidence functions (CIF) to account for competing risks.

Univariable and multivariable Cox regression models were estimated to assess the effect of vascular involvement on the outcomes of interest. Results were reported as Hazard Ratio (HR), 95% Confidence Interval (CI), and *p* value.

Univariable logistic regression was performed to assess the effect of vascular involvement on the site of recurrence. results were performed as Odds ratio (OR), 95% CI and *p* value.

Analyses were performed using R software (R Core Team, http://www.r-project.org/index.html) within rms, survival, and cmprsk packages.

## 3. Results

Between 1990 and December 2020, 465 patients underwent resection of thymic epithelial tumors at the Thoracic Surgery Unit of the University Hospital of Padua. Among these patients, 144 met the inclusion criteria and were eligible for our study.

The baseline characteristics of the population are reported in Table 1.

Pathological involvement of the great vessels was confirmed in 46 patients. In the vascular group there was a higher proportion of patients who underwent induction chemotherapy and who required a more invasive surgical approach. On the contrary, patients without vascular involvement were more frequently treated with postoperative chemotherapy.

There were no differences in terms of R0 resection rate, Masaoka stage distribution and WHO classification. Post-operative mortality was 2% in both groups: the two patients in the non-vascular group died of an acute myocardial infarction and a massive pulmonary embolism respectively, whereas the single patient in the vascular group died of multi organ failure 15 days after the operation.

Among patients with vascular involvement, in two patients the left innominate vein was resected without any further reconstruction, 12 patients underwent a tangential resection with direct reconstruction, six had a patch reconstruction and 26 had a prosthetic replacement.

Regarding this last subgroup, 10 patients had an isolated resection and reconstruction of the left innominate vein and one patient an isolated resection and reconstruction of the SVC. In 14 cases there was a complete resection of the SVC and of both veins: six patients had bilateral prosthetic reconstruction of the LIV and RIV, in four cases a single prosthetic bypass was placed between the SVC and the RIV and in 4 other cases between the SVC and the LIV. In one case the tumor invaded both the innominate veins and the brachiocephalic artery, therefore the reconstruction was performed using a double PTFE prosthesis between the innominate veins and the SVC, while the arteries were reconstructed with Dacron prosthesis.

Post-operatively, all patients were treated with low-molecular-weight heparin until complete mobilization and, in patients with prosthetic replacement, low-dose acetylsalicylic acid was administered lifelong.

During the follow-up, five patients with prosthetic replacement and one with patch reconstruction developed occlusion of the prosthesis or of the vessel. In three cases (two prosthetic replacement, one patch reconstruction) they developed a consequent edema of the upper limbs and were treated with anticoagulant therapy with a progressive resolution of the symptoms. No pulmonary embolism or other life-threatening events related to the vascular procedure were reported.

**Figure 2:**
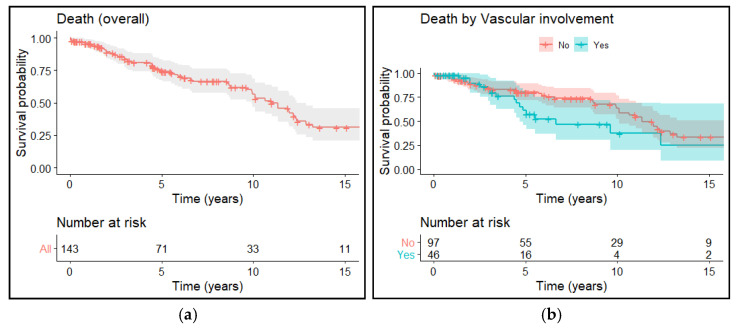
(**a**) Overall survival of the entire population; (**b**) Overall survival according to vascular and non-vascular involvement.

After a median follow-up of 4.9 years, 86 patients are still alive. The 5- and 10-years OS rates were 75% and 56%, respectively, with no differences between the two groups (*p* = 0.072) (Figure 2a,b).

**Figure 3:**
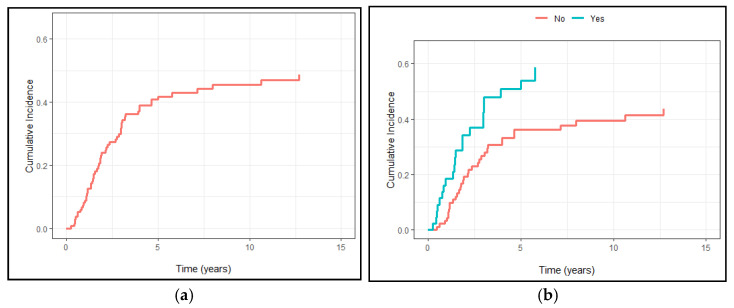
(**a**) Cumulative recurrence incidence in the entire population; (**b**) Cumulative recurrence incidence divided by vascular and non-vascular involvement.

Tumor recurrence was found in 57 patients, 22 in the vascular group and 35 in the other group (*p* = 0.2). The 5- and 10-years DFS rates were 59% and 54%, respectively (Figure 3a), whereas the correspondent DFS rates for the vascular and non-vascular groups were 49% vs. 63% and 41% vs. 60%, respectively, with a statistically significant difference among the two groups (*p* = 0.035) (Figure 3b).

**Figure 4:**
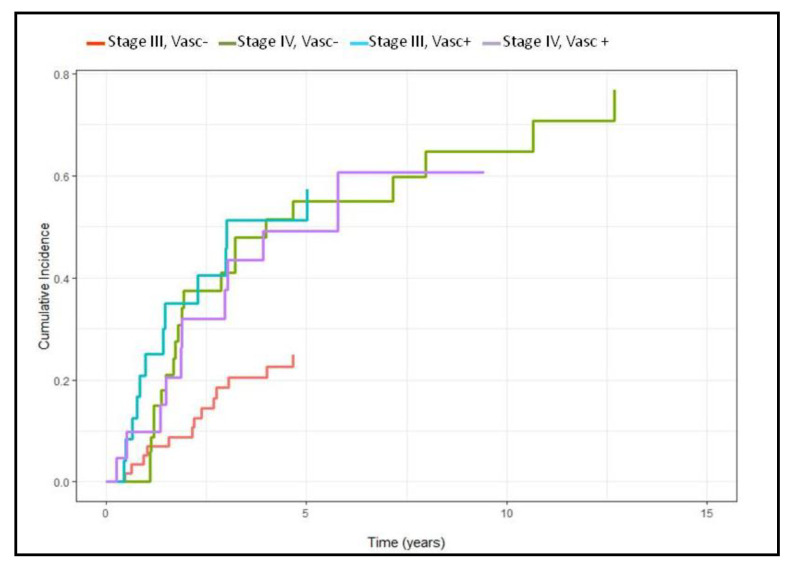
Cumulative recurrence incidence according to stage and vascular involvement.

When dividing the population according to the Masaoka stage and the vascular involvement there were also significant differences among the subgroups, with stage III disease without vascular involvement that showed a reduced incidence of developing recurrence, compared to stage III patients with vascular involvement (*p* = 0.003), with this latter subgroup showing an incidence of recurrence comparable with stage IV (Figure 4).

Regarding the site of recurrence, according to the ITMIG classification, in one case there was a local recurrence, in 18 cases a regional/pleural recurrence and in 26 a distant recurrence with no differences between the vascular and non-vascular group (*p* = 0.15).

In patients with distant recurrence, the most common site was the lung (13/26) followed by the liver (10/26). In 10 cases a pleural recurrence was associated, whereas in 13 cases (50%) the site of recurrence was multiple (Table 2).

At univariable analysis, recurrence was significantly associated to the vascular involvement (HR 1.7, 1.04–3.06, *p* = 0.035), WHO classification (thymic carcinoma vs. thymoma HR 2.8, 1.62–5.06, *p* < 0.001), Masaoka stage (HR 2.34, 1.33–4.10, *p* = 0.003), need for induction chemotherapy (HR 2.67, 1.53–4.66, *p* = 0.001), while R0 resection (HR 0.05, 0.01–0.23, *p* = <0.001) and myasthenia gravis (HR 0.32, 0.15–0.67, *p* = 0.003) were associated to a lower hazard of relapse.

R0 resection and WHO classification were the only two factors confirmed as a predictor for recurrence also at multivariable analysis (HR 0.11, 0.02–0.54, *p* = 0.006 and HR 2.27, 1.22–4.24, *p* = 0.01, respectively).

Concerning the analysis of the site of recurrence, the vascular involvement was not associated with a higher risk of distant metastasis (OR 2.22, 0.74–6.68, *p* = 0.15) and neither were thymic carcinomas compared to thymomas (OR 1.96, 0.62–6.27, *p* = 0.25).

However, when dividing the patients with distant metastasis according to the histotype there was a different distribution in the site of recurrence. Indeed, 11/16 (68%) patients with thymoma developed lung nodules, while only 2/10 (20%) with thymic carcinoma showed this site of recurrence (*p* = 0.006). On the contrary, although not significant, there was a higher incidence of liver and bones recurrence in the thymic carcinoma group (60% vs. 25%, *p* = 0.07, and 30% vs. 6%, *p* = 0.1, respectively).

## 4. Discussion

Infiltration of the mediastinal great vessels, particularly the innominate veins and the SVC, is a common finding in advanced TET. Vascular infiltration increases the technical complexity of the operation, requiring extended resections and demanding reconstructive procedures, with the aim of achieving a radical tumor resection. However, when performed in high-volume centers, these operations can be carried out with satisfactory outcomes.

Indeed, in our experience there was just one (2%) post-operative death in the patients undergoing vascular reconstructions, which is comparable to the main series reported in the literature (0–8%) [2,12,13,14,15,16,17], while the postoperative complication rate was 26%, but in only three cases related to the vascular procedure.

Notably, patients with vascular involvement were approached only through open approach because of the need of an extensive vascular dissection, but this did not influence the overall complication rate compared with patients in the non-vascular group.Regarding the best strategy for vessel replacement there are still many controversies. Indeed, we always used PTFE conduit to replace the venous vessels. Ringed PTFE grafts have been reported to persist patent for several years and to become re-epithelialized, having a low risk of infection and of less platelet deposition. Moreover, their rigidity prevents graft collapse when the central venous pressure becomes negative, avoiding compression caused by surrounding structures. On the other hand, the main problem of PTFE seems to be the need for long-term anticoagulation therapy and the risk of thrombosis compared to biological materials [2].

Notably, the optimal postoperative anticoagulant/antiplatelet therapeutic management in patients with PTFE conduit is not well-established. We routinely administer low-dose acetylsalicylic acid in all patients and reported five (19%) occlusion/sub-occlusion of the conduit that became clinically significant only in two cases; these data are comparable to the ones of the main series reported in the literature (8–50%) [2,12,13,14,15,16,17]. Particularly, Shintani and colleagues described an occlusion in up to 66% of patients with double-prosthesis reconstruction; indeed, also in our experience this subgroup showed the lowest patency rate (58%) [16].

Regarding the oncological point of view, the rarity of TET precludes well-powered prospective trials, therefore defined treatment strategies for advanced stage thymoma are unclear. It is well-established that surgical resection, alone or as one component of multimodality therapy, is the mainstay of thymoma treatment and can improve survival even in patients at an advanced stage [1,7,18]. Certainly, the aim of surgery is to obtain an R0-R1 resection, which is the most important prognostic factor, whereas patients undergoing debulking surgery show poor prognosis [19,20]. Chemotherapy and radiotherapy as definitive therapy are considered only in unresectable patients but with unfavorable outcomes [21].

On the contrary, resectability and survival may be improved with multimodality treatment (preoperative chemotherapy, surgery, and postoperative chemotherapy or radiotherapy) in patients with stage III and IV thymomas. Indeed, different experiences show that the ability to achieve a complete resection is higher after induction therapy, compared with rates in studies involving upfront surgery [20].

However, despite a complete surgical resection and multimodal therapies, recurrence rates are still high, with a reported incidence in the literature of about 10–40%, comparable to the data of this study (39.5%) [22,23,24]. It must be considered that we only analyzed patients with an advanced stage of the disease and also included thymic carcinomas, and who were therefore at a higher risk of developing a recurrence.

Large multicentric studies have allowed us to identify several predictors of recurrence, the most relevant being the completeness of surgical resection, the WHO classification and the Masaoka/IALSC stage [4,25]. In this context however, the prognostic role of the involvement of the great vessels is still unclear.

Okumura and colleagues first reported the vascular involvement as a negative prognostic factor in terms of survival and this observation was also confirmed by Tseng and colleagues [5,6,26].

Similarly, Utsumi and colleagues described a higher rate of recurrence (particularly systemic recurrence) in case of vascular involvement [27]. Moreover, the distant recurrence seemed to be less suitable for redo-operation and was therefore associated with a negative prognosis. Based on these observations, they speculated that tumor cells would pass through the superior vena cava, right atrium and ventricle, and pulmonary artery, before being scattered in the pulmonary capillaries and becoming implanted there to grow as a metastatic lesion.

On the contrary, in a large series such as the IASLC/ITMIG analysis, no clear differences were found relatively to the structures invaded in terms of the recurrence rate [4,28]. In the work by Yamada and colleagues, vascular invasion did not influence the rate of recurrence or the site of recurrence. Instead, patients with involvement of the vessels had lower R0 resection rate compared to pulmonary or pericardial invasion [29].

In our study, we found that the vascular involvement did not influence the overall survival, but this subgroup of patients had a higher recurrence rate, although no differences in terms of site of recurrence were reported.

Indeed, when feasible, surgical resection of recurrences, even reiterative, has been associated with favorable outcomes and this can explain the absence of differences in terms of overall survival. Moreover, the two groups did not differ in the proportion of patients who developed extrathoracic metastasis, which, differently from pleural or pulmonary recurrence, may preclude further surgical treatments.

Interestingly, when dividing the patients according to stage and vascular involvement, we showed that patient with stage III TET and vascular involvement had a risk of disease recurrence as high as patients with stage IV, therefore these patients should have a stricter follow-up or could benefit from more intense adjuvant treatments.

Regarding the site of recurrence, in terms of distant versus loco-regional, it was not influenced by vascular involvement and neither was by the disease histotype. However, the histotype seems to influence the site of the distant recurrence, with a higher rate of pulmonary metastasis in thymomas compared to thymic carcinoma, that instead showed a more frequent involvement of the bones and of the liver. The different biological behavior between thymomas and thymic carcinomas is well-known, and this different pattern of recurrence seems to underline different spreading ways. As previously said, pulmonary or pulmonary plus pleural recurrence seem to be more suitable for complete resection rather than extrathoracic sites, therefore these patients show better survival rates, as previously described by Sandri and colleagues and also confirmed by our data [30].

Interestingly, we noticed a different multimodal approach between the two groups, with patients with vascular involvement being treated more frequently with induction chemotherapy, whilst the non-vascular group showed a higher incidence of adjuvant treatments.

This may be explained by our preference, in case of preoperative suspicion of vascular involvement, to perform an adjuvant chemotherapic treatment in order to reduce the tumor’s dimension. This could increase the rate of complete resection, allow for a less demolitive operation, and thus possibly spare the vessel. Consequently, in these patients there are less possibilities to perform a chemotherapic treatment in the postoperative setting.

The main limitation of our study is its retrospective nature, thus allowing for selection biasassociated with the relatively small sample size. Furthermore, the patients were included over a long period of time, over 30 years, possibly affecting the different multimodal therapeutic approaches, particularly the chemotherapic approaches. Moreover, many patients were followed by other oncological centers, therefore indication for adjuvant or induction treatment may vary across the centers.

## 5. Conclusions

In conclusion our study demonstrates that the resection of thymic tumors with vascular involvement is a sound and relatively safe procedure when performed in a high-volume center, with optimal results also in the case of vascular reconstruction. From an oncological point of view, the involvement of the great vessels seems to be associated with a higher recurrence rate, but without affecting long term survival.

## Figures and Tables

**Table 1:** Baseline, operative and post-operative characteristics of the population.

	Total (*n* = 144)	Vascular Group ( *n* = 46)	Non-Vascular Group (*n* = 98)	*p*
Gender				0.538
− Male	76 (53%)	26 (57%)	50 (51%)	
− Female	68 (47%)	20 (43%)	48 (49%)	
Age, years, median (IQR)	56.2 (48–66)	54.3 (48–65)	56.4 (49–67)	0.5
Myasthenia Gravis	39 (27%)	6 (13%)	33 (34%)	0.009
Neoadjuvant CT	74 (51%)	37 (80%)	37 (38%)	<0.001
Neoadjuvant RT	6 (4%)	1 (2%)	5 (5%)	0.4
Surgical Approach				
− Sternotomy − Thoracotomy − Sternothoracotomy − Clamshell − VATS/Robotic	85 (59%)	26 (56.5%)	59 (60%)	0.014
	12 (9%)	0 (0%)	12 (13%)	
	38 (26%)	18 (39%)	20 (20%)	
	3 (2%)	2 (4.5%)	1 (1%)	
	6 (4%)	0 (0%)	6 (6%)	
R0 resection	126 (87.5%)	38 (82%)	88 (89%)	0.2
Tumor dimension, mm, median (IQR)	80 (57–100)	75 (60–100)	80 (56–100)	0.78
Post-operative complications	33 (23%)	12 (26%)	21 (21%)	0.53
Post-operative deaths	3 (2%)	1 (2%)	2 (2%)	0.98
Pathological Masaoka Stage				
− III − IVa − IVb	86 (60%)	25 (55%)	61 (62%)	0.1
	44 (30.5%)	13 (28%)	31(32%)	
	14 (9.5%)	8 (17%)	6 (6%)	
WHO Classification				
− A − AB − B1 − B2 − B3 − C	4 (3%)	2 (4%)	2 (2%)	0.15
	12 (8.5%)	1 (2%)	11 (11%)	
	21 (14.5%)	6 (13%)	15 (15%)	
	36 (25%)	12 (27%)	24 (25%)	
	42 (29%)	11 (25%)	31 (32%)	
	29 (20%)	14 (29%)	15 (15%)	
Adjuvant CT	22 (14%)	2 (4%)	20 (20%)	0.01
Adjuvant RT	106 (74%)	33 (72%)	73 (74%)	0.7
Recurrence	57 (39.5%)	22 (49%)	35 (36%)	0.15
− Local	1 (1%)	0 (0%)	1 (3%)	
− Regional	30 (53%)	9 (41%)	21 (60%)	
− Distant	26 (46%)	13 (59%)	13 (37%)	

**Table 2:** Characteristics of the patients with distant recurrence.

	Histology	Pleura	Lung	Liver	Bone	Lymp Nodes	Other
Vascular Group							
− Case 1	Thymic Carc.			+			
− Case 2	Thymoma		+				
− Case 3	Thymic Carc.			+	+		
− Case 4	Thymoma	+	+				
− Case 5	Thymic Carc.			+			
− Case 6	Thymoma	+				+	
− Case 7	Thymoma		+				
− Case 8	Thymoma	+	+				
− Case 9	Thymic Carc.						+
− Case 10	Thymoma			+			
− Case 11	Thymoma		+				
− Case 12	Thymic Carc.	+	+	+			
− Case 13	Thymoma	+	+	+			
Non Vascular Group							
− Case 1	Thymoma						+
− Case 2	Thymoma		+				
− Case 3	Thymoma	+	+				+
− Case 4	Thymoma	+				+	
− Case 5	Thymoma		+			+	
− Case 6	Thymic Carc.	+		+			
− Case 7	Thymic Carc.				+		
− Case 8	Thymic Carc.		+			+	
− Case 9	Thymoma		+				
− Case 10	Thymoma	+	+	+			
− Case 11	Thymic Carc.	+			+		
− Case 12	Thymic Carc.			+			
− Case 13	Thymoma			+			

## Data Availability

The data presented in this study are available on request from the **c**orresponding authors.
